# Supplementary figures and images for: Insights on the historical biogeography of Philippine domestic pigs and its relationship with continental domestic pigs and wild boars
Source: PLoS One. 2022 Mar 28;17(3):e0254299. doi: 10.1371/journal.pone.0254299 (PMC8959178; doi:10.1371/journal.pone.0254299)

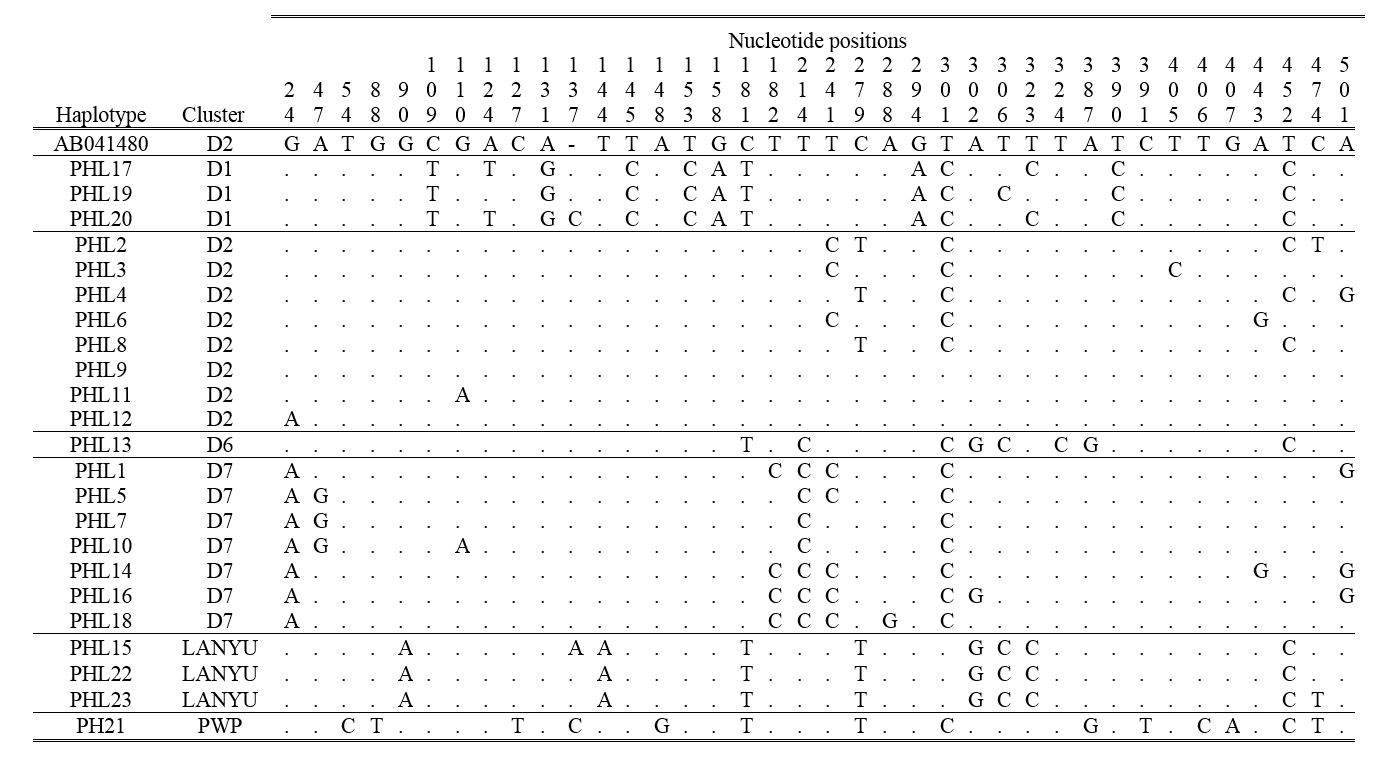

Supplement: S1 Fig — Dots (.) indicates matches with the nucleotide sequence GenBank accession number AB041480 (Main cluster of Asian origin). PWP = Philippine wild pigs; D7 = previously described as MTSEA haplogroup. Nucleotide positions are numbered according to our sequence alignment. (TIF) [file pone.0254299.s001.tif]

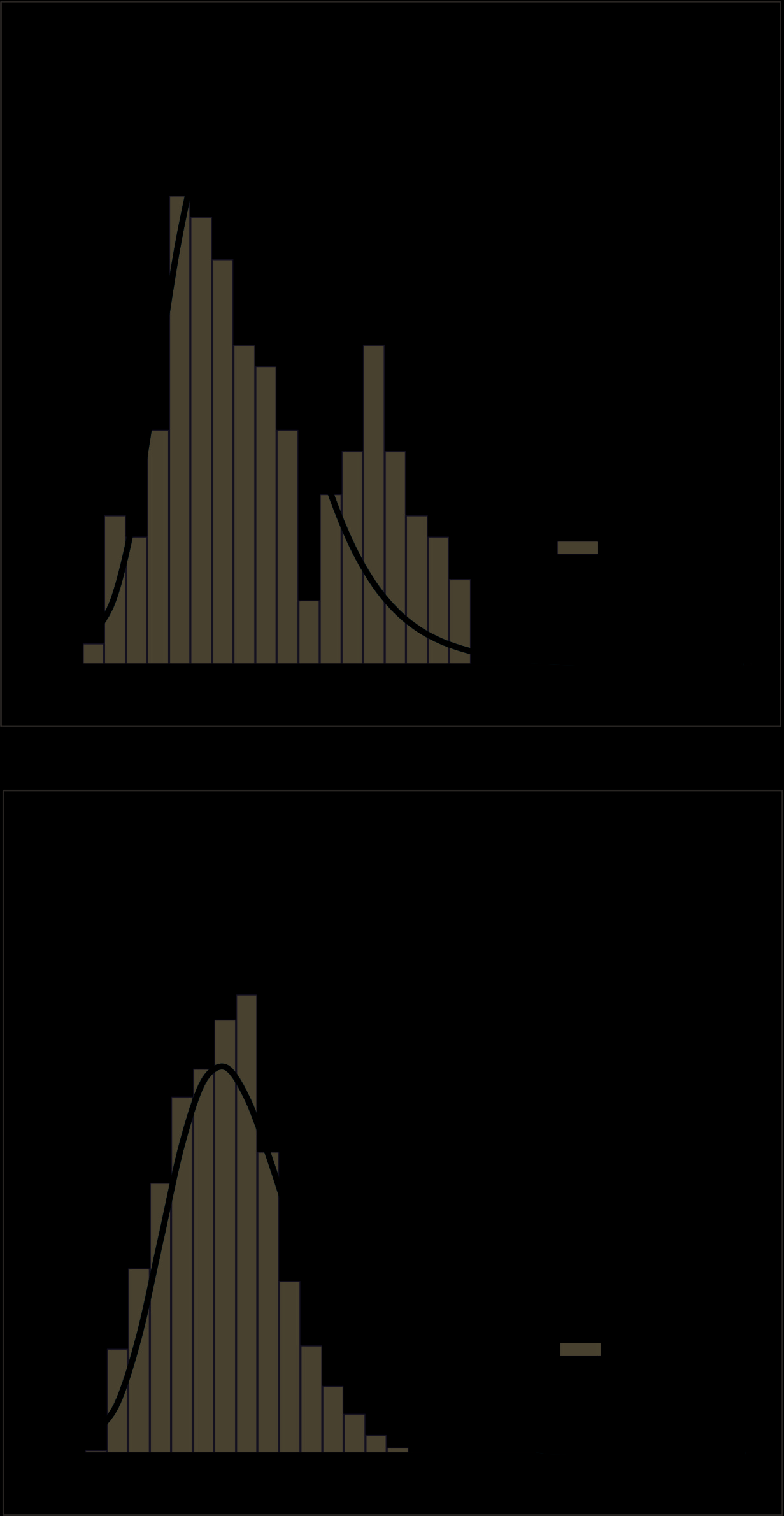

Supplement: S2 Fig — Mismatch distributions of mitochondrial DNA sequences of the (A) Philippine domestic pigs, (B) mainland SEA pigs based on pairwise nucleotide differences. (TIF) [file pone.0254299.s002.tif]

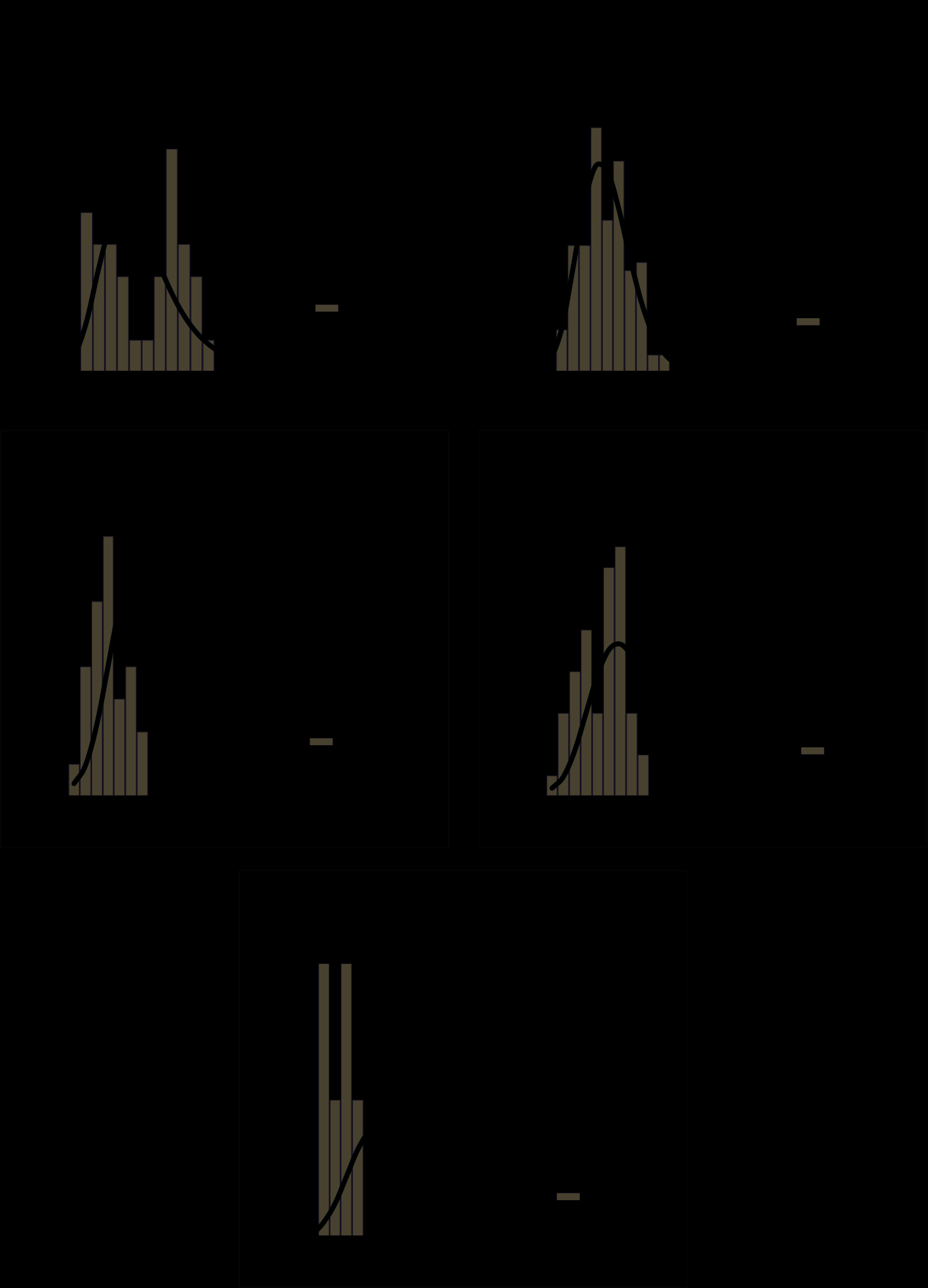

Supplement: S3 Fig — (TIF) [file pone.0254299.s003.tif]

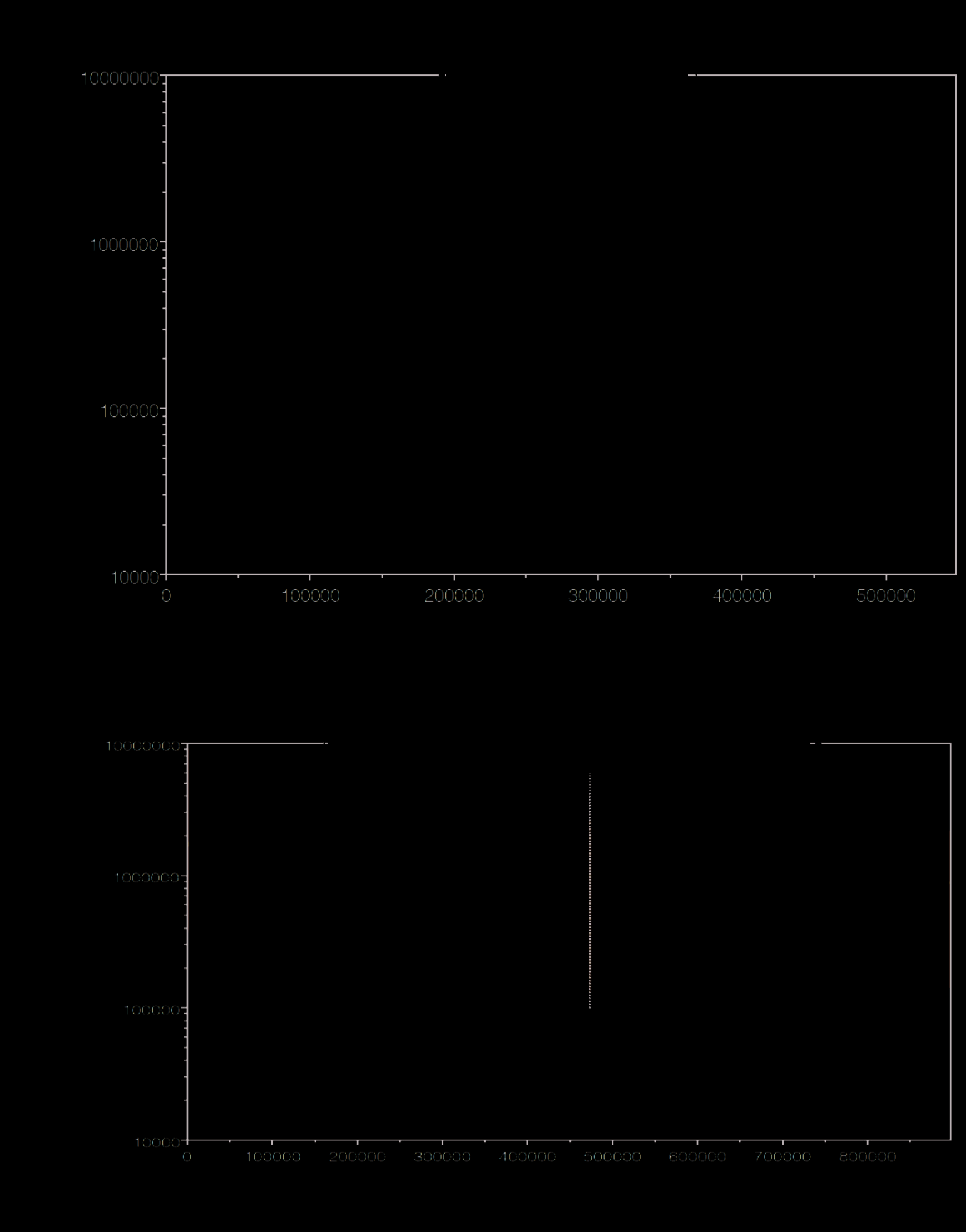

Supplement: S4 Fig — Bayesian skyline plots showing effective population size of (A) Philippine and (B) mainland Southeast Asian pigs. Median estimates of female effective population size (Nef) are shown as solid thick line (blue) and the light-blue shaded area marks the 95% credibility intervals. The abscissa is scaled in thousands of years before present (BP). The Philippine pigs revealed a long stationary period of effective population size and the population decrease event occurred roughly at about ~25,000 BP. (TIF) [file pone.0254299.s004.tif]

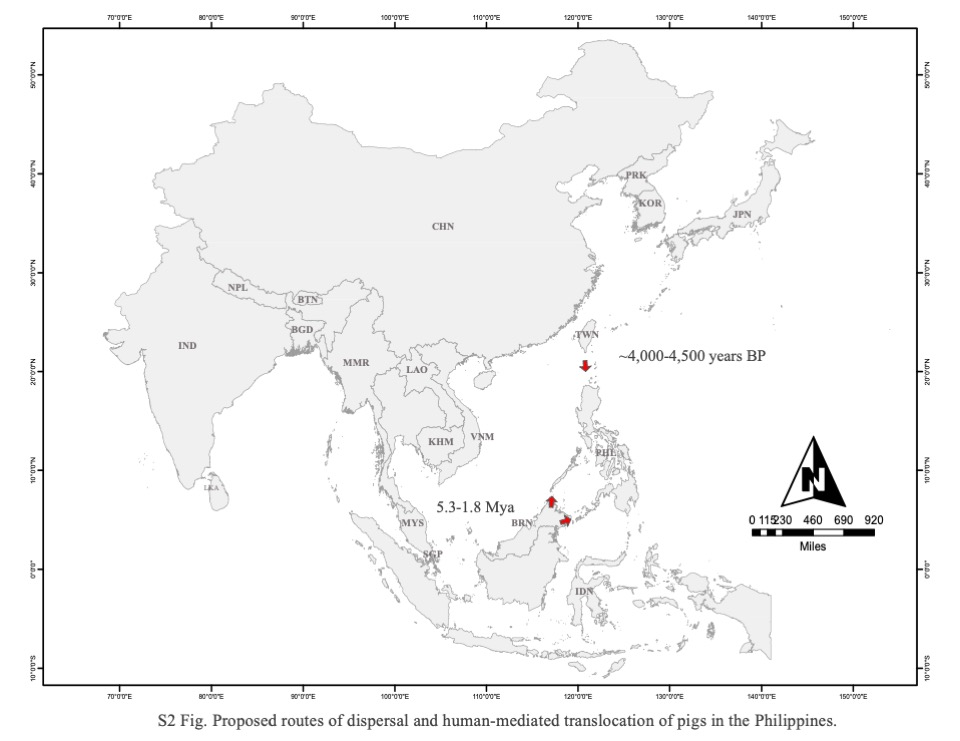

Supplement: S5 Fig — (TIF) [file pone.0254299.s005.tif]
