# Supplementary material for: Insights on the historical biogeography of Philippine domestic pigs and its relationship with continental domestic pigs and wild boars
Source: PLoS One. 2022 Mar 28;17(3):e0254299. doi: 10.1371/journal.pone.0254299 (PMC8959178; doi:10.1371/journal.pone.0254299)
Supplement: Attachment — Submitted filename: Response to Reviewers.docx [file pone.0254299.s009.docx]

Reviewer #1: The subject manuscript reports on a study evaluating the origin, dispersal, and level of genetic introgression in Philippine “native” pigs using mitochondrial DNA D-loop analysis. These genetic data are then compared to comparable mtDNA data from domestic pigs and Eurasian wild boar. While the stated goal of the study is straightforward, the exact composition of the pigs being investigated is not completely clear.

There are no “native” *Sus scrofa*, either wild or domestic, in the Philippines. Meijaard and Melletti (2018) list the endemic (“native”) wild pigs that exist in the Philippine archipelago as including the following: Visayan warty pig (*Sus cebifrons*), Philippine warty pig (*Sus philippensis*), Mindoro warty pig (*Sus oliveri*), and Palawan bearded pig (*Sus ahoenobarbus*). Fossils of these Philippine wild pig species variously date as far back as the Pliocene through the late Pleistocene. The earliest documented archaeological remains of domestic pigs belonging to the species *Sus scrofa* in the Philippines were from a site in the Cagayan Valley dating back to 4000 BP (Heaney et al. 2016). Hybridization between domestic pigs (*Sus scrofa*) and Visayan warty pigs (Melletti et al. 2018), Philippine warty pigs (Meijaard and Melletti 2018), and Mindoro warty pigs (Tabaranza et al. 2018) in the Philippines has been documented. More specifically, such hybridization between domestic/feral *Sus scrofa* and Philippine warty pigs has been observed on Luzon, Mindanao, Basilan and other islands in the archipelago (Heaney et al. 2005).

We have added information in the introduction about the numerous endemic Philippine wild pigs, as well as the hypothesized introduction of domestic pig in the Philippines.

The following citations were also added:

1. Oliver WLR. The taxonomy, distribution and status of Philippine wild pigs. Ibex, Journal of Mountain Ecology. 1995;3: 26–32.
2. Luskin MS, Ke A. Bearded pig *Sus barbatus* (Müller,1838). In: Meletti M, Meijaard E, editors. Ecology, Conservation and Management of Wild Pigs and Peccaries. Cambridge University Press; 2018. pp. 176.
3. Piper PJ, Hung HC, Campos FZ, Bellwood P, Santiago R. A 4000-year-old introduction of domestic pigs into the Philippine archipelago: Implications for understanding routes of human migration into through Island Southeast Asia and Wallacea. Antiquity. 2009;83: 687–695.
4. Amano N, Piper PJ, Hung HC, Bellwood P. Introduced domestic animals in the Neolithic and Metal Age of the Philippines: Evidence from Nagsabaran, Northern Luzon. J. Island Coast. Archaeol. 2013. 8, 317-335.

All of that said, it solicits the question as to what were the pigs that this study was looking at. Given the comparisons to genetic data from known breeds of domestic pigs and Eurasian wild boar, the study would seem to be looking at only the introduced domestic pigs belonging to the species *Sus scrofa*. To that end, the manuscript needs to not use the modifier “native” for these domestic pigs, and these pigs should be specifically identified as “domestic” (or “local domestic”, “rural domestic”, or “local rural domestic” or something like that; the modifier “native” is misleading). On Line 113, it was noted that samples were also collected from Philippine “wild pigs”. Further, ID sample numbers P24 and P25 in Table S1 are listed as wild pigs from the Philippines. Accession No. MN625830 in Table S2 is listed as a “Philippine wild pig.” What were these Philippine wild pigs, feral *Sus scrofa*? There are no Eurasian wild boar present in the Philippines. Were these “wild pigs” in actuality warty pigs and not feral *Sus scrofa*? The two pigs illustrated in Fig. 1 A and G appear to be immature pure or hybrid warty pigs and not *Sus scrofa*. In a number of places in the manuscript the authors refer to “pigs”. This noun should be more specifically defined (e.g., “pigs, which includes both wild and domestic pigs” or “pigs, which includes only domestic pigs”). In most of these places, the reader is left guessing as to what types of pigs are being discussed. A single explanatory statement on this can be made in the introduction, which would then clarify the subsequent use of the noun “pig.” The authors also need to address the potential impacts of genetic introgression of warty pigs through hybridization with domestic *Sus scrofa* in the Philippines on their results. Is this something that was even considered in the analysis of the data?

We would like to apologize for the confusion in the modifiers used in the manuscript. We have changed all modifiers "native pig" to "domestic pig" throughout the manuscript where appropriate to avoid further confusion. The Philippine wild pig described in this study is indeed a wild pig. In this case, we did not use the term "warty pigs" in the discussion because the classification of the wild pig analyzed is not yet known. Therefore, to be on the safe side, we use the term "wild pigs" throughout the dataset. Figures 1A and B are Philippine domestic pigs, not warty pigs. As a future direction for this research, we intend to analyze the potential impact of wild pigs on domestic pigs in the Philippines, as we did not conduct such an analysis in this study.

To my knowledge, this is the first study that has specifically looked at genetic composition as related to the anthropogenic origin of domestic pigs in the Philippines. As such, the data encompassed by the subject manuscript are both unique and important. Overall, the manuscript is well written. The editorial comments noted below are largely intended as suggested changes and should be used by the authors as deemed appropriate:

We appreciate your positive feedback on our study. Yes, this study is the first screening of mtDNA D-loop variation from Philippine domestic pigs to clarify their distribution and past dispersal history. In the future, the authors would like to conduct further relevant research in this field to bridge the gap between the highly controversial anthropogenetic causes of diversity in the Philippines, especially in domestic pigs. We believe that this publication is a start so that we can attract future collaborators for a larger research project.

Line 1 – Does “Philippine native pigs” refer to wild or domestic pigs? There are no “native” domestic pigs in the Philippine islands.

Revised accordingly. We changed the modifier “native pigs” to “domestic pigs”.

Line 39 – Suggest changing “native” to “domestic”

Revised accordingly (see Line 46 in the revised manuscript).

Line 45 – Does this refer to domestic pigs or both domestic and wild pigs?

We are referring to Philippine domestic pigs. Changed to Philippine domestic pigs as suggested.

Line 51 - Does this refer to domestic pigs or both domestic and wild pigs?

The authors are referring to Philippine domestic pigs. Changed to Philippine domestic pigs as suggested.

Line 77 - I assume that this is referring to wild *Sus scrofa* (Yes?)

Yes.

Line 78 - Suggest changing “exists” to “exist”; as a species, *Sus scrofa* would be a plural subject.

Revised accordingly.

Lines 78-79 - Does “S. scrofa that exists in the Philippines today” mean the wild/feral or domestic *Sus scrofa* that exist in the Philippines today?

This phrase has been changed to “Therefore, the ubiquitous wild boar *S. scrofa* was not considered native to the Philippines because it was unable to reach the archipelago [15-19] and was probably introduced as a domestic animal within the last few thousand years [19]” (see Lines 94-96 in the revised manuscript).

Lines 96-97 – Does “some modern and ancient Philippine pigs” refer to wild or domestic pigs or both?

The authors are referring to Philippine domestic pigs (see Lines 116-118 in the revised manuscript).

Line 99 – Suggest changing “pigs” to “domestic pigs”

Revised accordingly (see Line 119 in the revised manuscript).

Line 103 - Suggest changing “Philippine pigs” to “Philippine domestic pigs”

Revised accordingly (see Line 132 in the revised manuscript).

Line 113 – Referring to “Philippine native pigs”, does this mean domestic pigs? If so, then use the modifier domestic and not native. Looking in the Supplemental tables, this must mean domestic pigs.

Revised accordingly (see Line 143 in the revised manuscript).

Line 139 – Suggest changing “Philippine native pigs” to “Philippine domestic pigs”

Revised accordingly (see Line 177 in the revised manuscript).

Line 177 – Does “Philippine pigs” refer to wild or domestic pigs or both?

Both. (see Line 216 in the revised manuscript)

Line 196 – Remove the extra space between “test” and “[39]”

Revised accordingly (see Line 235 in the revised manuscript).

Line 219 – Suggest changing “Philippine native pigs” to “Philippine domestic pigs”

Revised accordingly (see Line 258 in the revised manuscript).

Line 230 – The “native and domestic pigs” are both domestic pigs (Yes?)

Yes. “native and” was deleted (see Line 269 in the revised manuscript).

Line 238 - Suggest changing “Philippine native pigs” to “Philippine domestic pigs”

Revised accordingly (see Line 277 in the revised manuscript).

Line 244 – Insert a comma after “0.01)” (0.01), which)

Revised accordingly (see Line 283 in the revised manuscript).

Line 254 - Suggest changing “Philippine native pigs” to “Philippine domestic pigs”

Revised accordingly (see Line 293 in the revised manuscript).

Line 261 - Suggest changing “Philippine native pigs” to “Philippine domestic pigs”

Revised accordingly (see Line 300 in the revised manuscript).

Line 272 - Suggest changing “Philippine native pigs” to “Philippine domestic pigs”

Revised accordingly (see Line 311 in the revised manuscript).

Line 272-273 – Should “Asian and European pigs” be changed to “Asian and European domestic pigs” or does that sample also include data from Eurasian wild boar?

This samples include Eurasian wild boars (see Lines 311-312 in the revised manuscript).

Lines 293-299 - Could the animal discussed in this portion of the text in reality have been a warty pig and not a wild/feral *Sus scrofa*? This species difference might explain these genetic variances. Where is this animal (PHL21) listed in the Supplementary tables?

This animal is actually a warty pig and not a feral/wild *Sus scrofa*. However, in this case we used the general term "Philippine wild pigs" for this haplotype because its species classification has not yet been identified (see Line 331 in the revised manuscript). This haplotype/sample can be found in S1 Table ID #P24&25, S2 Table #67 under accession number MN625830.

Line 301 - Suggest changing “Philippine native pigs” to “Philippine domestic pigs”

Revised accordingly (see Line 339 in the revised manuscript).

Line 302 – Suggest changing “continental pigs and wild boar” to “continental domestic pigs and wild boar”

Revised accordingly (see Line 340 in the revised manuscript).

Line 304 – Suggest changing “Philippine pigs” to “Philippine domestic pigs” unless this includes both domestic and wild animals.

Revised accordingly (see Line 342 in the revised manuscript).

Line 306 – What does “endemic Philippine wild pigs” refer to? Feral *Sus scrofa*? Native/endemic warty and/or bearded pigs?

The authors are referring to endemic Philippine wild pigs (see Line 343 in the revised manuscript).

Line 337 – Suggest changing “Philippine native pigs” to “Philippine domestic pigs”

Revised accordingly (see Lines 374-375 in the revised manuscript).

Line 339 - Suggest changing “Philippine native pigs” to “Philippine domestic pigs”

Revised accordingly (see Line 376 in the revised manuscript).

Line 358 - Suggest changing “Philippine native pigs” to “Philippine domestic pigs”

Revised accordingly (see Line 396 in the revised manuscript).

Lines 369-370 - Suggest changing “Philippine native pigs” to “Philippine domestic pigs”

Revised accordingly (see Lines 407-408 in the revised manuscript).

Line 382 - Suggest changing “Philippine native pigs” to “Philippine domestic pigs”

Revised accordingly (see Line 411 in the revised manuscript).

Line 384 – The acronym “NEA” needs to be defined.

Revised accordingly (see Line 413 in the revised manuscript).

Line 385 – Suggest changing “Philippine pig genetic pool” to “Philippine domestic pig genetic pool” unless this includes both domestic and wild animals.

Revised accordingly (see Line 414 in the revised manuscript).

Line 409 - Suggest changing “Philippine pig genetic pool” to “Philippine domestic pig genetic pool”

Revised accordingly (see Line 440 in the revised manuscript).

Line 410 - Suggest changing “Philippine native pigs” to “Philippine domestic pigs”

Revised accordingly (see Line 441 in the revised manuscript).

Line 411 – Suggest changing “Chinese pigs” to “Chinese domestic pigs”

Revised accordingly (see Line 442 in the revised manuscript).

Line 413 – Suggest changing “Philippine native pig” to “Philippine domestic pig”

Revised accordingly (see Line 444 in the revised manuscript).

Lines 414-415 - Suggest changing “Philippine native pigs” to “Philippine domestic pigs”

Revised accordingly (see Lines 445-446 in the revised manuscript).

Line 418 – Suggest changing “domestic and native pigs” to “domestic pigs”

Revised accordingly (see Lines 447 in the revised manuscript).

Line 422 - Suggest changing “Philippine native pigs” to “Philippine domestic pigs”

Revised accordingly (see Line 453 in the revised manuscript).

Lines 459-462 - Were the wild pigs referred to in this portion of the text referring to warty and/or bearded pigs or feral *Sus scrofa*?

The authors are referring to Philippine wild pigs (see Line 490 in the revised manuscript). All endemic wild pigs in the Philippines are commonly referred to as Philippine wild pigs unless genetically characterized.

Line 465 – Suggest changing “Philippine native pigs” to “Philippine domestic pigs”

Revised accordingly (see Line 496 in the revised manuscript).

Line 467 - Suggest changing “Philippine native pig” to “Philippine domestic pig”

Revised accordingly (see Line 498 in the revised manuscript).

Line 470 - Suggest changing “Philippine native pigs” to “Philippine domestic pigs”

Revised accordingly (see Line 501 in the revised manuscript).

Line 485 – Suggest changing “Philippine native pig” to “Philippine domestic pig”

Revised accordingly (see Line 516 in the revised manuscript).

Line 488 - Suggest changing “Philippine native pigs” to “Philippine domestic pigs”

Revised accordingly (see Line 519 in the revised manuscript).

Line 496 – Suggest changing “Philippine native pig” to “Philippine domestic pig”

Revised accordingly (see Line 524 in the revised manuscript).

Line 501 - Suggest changing “Philippine native pigs” to “Philippine domestic pigs”

Revised accordingly (see Line 529 in the revised manuscript).

Line 547 - Suggest changing “Philippine native pigs” to “Philippine domestic pigs”

Revised accordingly (see Line 573 in the revised manuscript).

Line 593 – Change “2017” to “2018”; the copyright date of this book is 2018. Look at the page after the title page in the book.

Revised accordingly (see Line 617 in the revised manuscript).

Line 607 – “*Sus*” needs to be italicized

Revised accordingly (see Line 637 in the revised manuscript).

Line 630 - “*Sus*” needs to be italicized

Revised accordingly (see Line 662 in the revised manuscript).

Line 699 - “*Sus scrofa*” needs to be italicized

Revised accordingly (see Line 640 in the revised manuscript).

Line 712 - “*Sus*” needs to be italicized

Revised accordingly (see Line 731 in the revised manuscript).

Lines 764-765 - “*Sus scrofa*” needs to be italicized

Revised accordingly (see Lines 778-779 in the revised manuscript).

References:

Heaney, L. R., D. S. Balete, G. V. Gee, et al. 2005. Preliminary report on the mammals of Balbasang, Kaling Province, Luzon. Sylvatrop: The Philippine Forest Research Journal 13:51-62.

Heaney, L. R., D. S. Balete and E. A. Rickart. 2016. The mammals of Luzon Island: biogeography and natural history of a Philippine fauna. Johns Hopkins University Press, Baltimore, Maryland, USA. 304 pp.

Melletti, M., and E. Meijaard (editors). 2018. Ecology, Evolution and Management of Wild Pigs and Peccaries: Implications for Conservation. Cambridge University Press, United Kingdom. 480 pp.

Meijaard, E., and M. Melletti. 2018. Philippine warty pig *Sus philippensis* (Nehring, 1886). pp. 157-161. in Ecology, Evolution and Management of Wild Pigs and Peccaries: Implications for Conservation. (M. Melletti and E. Meijaard, eds.). Cambridge University Press, United Kingdom. 480 pp.

Melletti, M., E. Meijaard, and L. Przybylska. 2018. Visayan warty pig *Sus cebifrons* (Heude, 1888). pp. 150-156. in Ecology, Evolution and Management of Wild Pigs and Peccaries: Implications for Conservation. (M. Melletti and E. Meijaard, eds.). Cambridge University Press, United Kingdom. 480 pp.

Tabaranza, D. G. E., E. Schütz, J. C. T. Gonzalez, L. M. Espiritu-Afuang. 2018. Mindoro warty pig *Sus oliveri* (Groves, 1997). pp. 162-169. in Ecology, Evolution and Management of Wild Pigs and Peccaries: Implications for Conservation. (M. Melletti and E. Meijaard, eds.). Cambridge University Press, United Kingdom. 480 pp.

Reviewer #2: The authors produce some interesting results that should be of interest to PLOS ONE readership. It is original research that has been ethically conducted with robust statistical methods. They contributed their genetic samples to GenBank and provided all of their supplemental materials for download. I believe if someone was so inclined they would be able to reproduce their research which is the hallmark of sound science.

The only thing that sticks out to me is that English is not their first language. There are no outright errors, it just doesn't flow well. If they could find a native English speaker to polish it a bit then it would be fantastic. My recommendation is a minor revision.

Thank you for your positive feedback on our paper and your recommendation for a minor revision. We have resubmitted our paper for English critique and proofreading to improve the manuscript. As you could notice, there were changes throughout the manuscript.

Reviewer #3: This manuscript is an important contribution to the discussion of human and human-mediated plant/animal global dispersion. I feel that the manuscript could benefit from some re-organization, refinement, and a bit more detail in the introduction.

Line 48 - What is the D7 haplogroup? I’m not sure the authors can refer to this without describing what it is.

Revised accordingly. We added some information about the D7 haplogroup in the Abstract (see Lines 54-55 in the revised manuscript).

LINE 50 - What is the significance of a D2 haplogroup how would the reader know what these are?

We have added quotation of D2 haplotypes in the Abstract "Asian major" (see Line 58 in the revised manuscript). Due to the limited number of words in the abstract, see Lines 323-325 and 458-461 of the revised manuscript for more details on this haplogroup. The phrase “which postulate the legitimate dispersal of pigs associated with the multiple waves of human migrations involving the Philippines” was deleted and was replaced by phrase “on several Philippine islands”.

Line 54-56 - It seems like a sentence may be missing or the phrasing is off. Perhaps another sentence concluding the significance of the work is needed.

Revised accordingly. We have also added the concluding remarks in the abstract: " This finding highlights an important challenge in managing Philippine domestic pig genetic resources from a local and global perspective" (see Lines 62-64 in the revised manuscript).

Introduction:
- The authors should consider breaking the introduction up into more paragraphs to help the reader.

Revised accordingly. We have revised almost the entire introduction (see Lines 67-136 in the revised manuscript).

-The authors should also consider adding in some discussion of the D-type haplogroups as it plays a very important role later on in the manuscript but is not described at all until the Discussion.

Revised accordingly. We have added discussions of the various haplogroups in the introduction (see Lines 78-85 in the revised manuscript).

- For example, lines 370-378 may be more appropriate for the introduction so the reader has some sense as to why, in the results section, there is reference to “D-type” haplotypes.

Revised accordingly (see Lines 78-85 in the revised manuscript).

- Furthermore, which D cluster refers to Asian Phylogeographic vs. European?? (See line 380)

By and large, the *Sus scrofa* was historically domesticated from two ancestral animals: the Asian and the European *Sus scrofa* wild boar. Later, the lineages of these wild boars separated, resulting in an independent evolutionary lineage known as "haplogroups." Today, the wild boars of Asian origin that are widely recognized are the D2, D3, D5, D6, D7 and the Lanyu pigs (Asian phylogeographic origin), while the European wild boars are the D1 and D4 (European phylogeographic origin) (see Fig 3).

- Line 222 - S4 appendix does not appear in the manuscript that the review has access to.

We apologize. S4 appendix refers to S1 Fig.

- Line 276 - It is not clear to me in Fig 2 the two distinct core lineages. Suggest that the authors revise Fig 2 to clearly indicate the “Asian” and “European” core lineages.

We revised accordingly. We have also decided to re-analyze this figure. We have changed our methodology in constructing the phylogenetic tree from Maximum Likelihood to Bayesian Inference using program Mr Bayes. Please see Fig 3 in the revised manuscript.

- Line 281 - The authors make it very difficult to follow the groupings by creating multiple different short-hand methods to describe geographic groupings. In line 281, the authors refer to MTSEA as D7. Suggest sticking to one of these classifications.

Revised accordingly (see Line 320 in the revised manuscript).

- S2 Fig - It would be helpful to have the countries labelled in this map so that the reader can refer to their specific locations when reviewing Table 1 as well as the introduction, specifically lines 78-86

Revised accordingly.

- Line 384 - What is “NEA”? I don’t see it defined anywhere else. The number of acronyms and confusing usage of them has mad reading this manuscript very challenging. Strongly suggest removing all acronyms or simplifying significantly.

Revised accordingly (see Line 413 in the revised manuscript).

- Line 397 - Perhaps the authors should consider introducing the importance of the “D” haplotypes to their results in the introduction so that the reader may understand the classification.

Revised accordingly (see Lines 78-85 in the revised manuscript).

- Line 393-398 - Seems like a key finding of the study, but it is buried in the middle of a large paragraph.

Revised accordingly. We have decided to separate this paragraph for emphasis. We have also added citation “The complexity surrounding the Neolithic Austronesian expansion and dispersal has recently raised questions due to the limited studies conducted in the Philippine archipelago, as the faunal assemblages in this key region have provided some clues [4]. For instance,” (see Lines 426-436 in the revised manuscript).

1. Larson G, Dobney K, Albarella U, Fang M, Smith EM, Robins J, et al. Worldwide phylogeography of wild boar reveals multiple centers of pig domestication. Science. 2005;307: 1618–1621.

- Perhaps the map could also include the geographic origins of the D-type haplotype clusters. The phylogenetic tree might also.

Revised accordingly. We have added figure on this. Please see Figure 1 in the revised manuscript.

- I’m having some trouble understanding how the Philippine “native” pigs that were presumably dispersed by humans (whether from Taiwan or otherwise) can be considered “native” and would want the authors to define what they consider to be native and at what point of genetic mixing would the animal no longer be classified as native? Further, in line 459 the authors refer to “wild” Philippine pigs. Are these different than “native” pigs?

We apologize for the confusion in the modifiers used. To avoid it, we have replaced all modifiers "native" with "domestic" throughout the manuscript where appropriate. Indeed, line 459 refers to the Philippine wild pig (endemic to the Philippines), whereas "native/domestic pigs" in this study refers to the introduced pig (see Line 490 in the revised manuscript).

- The authors might consider bringing an abridged summary of lines 443-458 into the introduction to further help the reader understand the context of this manuscript.

Revised accordingly. We have added additional citations in the introduction (see Lines 94-96, 101-103).
