# Supplementary material for: Insights on the historical biogeography of Philippine domestic pigs and its relationship with continental domestic pigs and wild boars
Source: PLoS One. 2022 Mar 28;17(3):e0254299. doi: 10.1371/journal.pone.0254299 (PMC8959178; doi:10.1371/journal.pone.0254299)
Supplement: Attachment — Submitted filename: Response to Reviewers 2.docx [file pone.0254299.s010.docx]

First and foremost, we appreciate for considering our manuscript for publication and to the reviewers for their valuable comments and suggestions for improvement. The reviewers' comments indeed helped us to improve the readability and quality of the manuscript. Thank you so much.

Reviewer #1: The subject revised manuscript reports on a study evaluating the origin, dispersal, and level of genetic introgression in Philippine domestic pigs using mitochondrial DNA D-loop analysis. These genetic data are then compared to comparable mtDNA data from domestic pigs and Eurasian wild boar. This revision is much improved over the earlier draft. Again, to my knowledge, this is the first study that has specifically looked at genetic composition as related to the anthropogenic origin of domestic pigs in the Philippines. As such, the data encompassed by the subject manuscript are both unique and important. Overall, the manuscript is well written. The editorial comments noted below are largely intended as suggested changes and should be used by the authors as deemed appropriate:

• Lines 43-44 – Suggest changing “the history of pig dispersal” to “the history of domestic pig (Sus scrofa) dispersal”.

Revised accordingly.

• Line 45 – Suggest changing “associated with some human migration events” to “associated with human migration events

Revised accordingly.

• Line 47 – Suggest changing “wild boars” to “wild boar”

Revised accordingly. Please see Lines 47-48 in the revised manuscript.

• Line 55 – Suggest changing “Philippine pig haplotypes” to “Philippine domestic pig haplotypes”

Revised accordingly.

• Line 67 – Suggest changing “The wild boar (Sus scrofa L.)” to “The Eurasian wild boar (Sus scrofa L.)”

Revised accordingly.

• Lines 86-87 – Suggest changing “an exceptionally high levels of endemism” to “an exceptionally high level of endemism”

Revised accordingly.

• Line 87 – Suggest changing “The Philippines has repeatedly” to “The Philippines have repeatedly”

Revised accordingly.

• Line 89 - Suggest changing “four endemic wild pigs such as the Philippine” to “four endemic wild pigs including the Philippine”

Revised accordingly.

• Line 91 – Suggest changing “as well as one native shared with Sundaic” to “as well as one native species shared with the Sundaic”

Revised accordingly.

• Line 94 – Suggest changing “ubiquitous wild boar S. scrofa was not considered native” to “ubiquitous Eurasian wild boar S. scrofa is not native”

Revised accordingly.

• Lines 111-112 – Suggest changing “For this reason, [27] excluded” to “For this reason, Larson et al. [27] excluded”

Revised accordingly.

• Line 128 – Suggest changing “an introduced pig breed” to “an introduced domestic pig breed”

Revised accordingly.

• Line 133 - Suggest changing “diversity and structure exhibits a signature” to “diversity and structure exhibit a signature”

Revised accordingly.

• Line 217 - Suggest changing “described by [4] with six clades” to “described by Larson et al. [4] with six clades”

Revised accordingly. Please see Lines 216-217 in the revised manuscript.

• Line 218 – Suggest changing “proposed by [10] were used” to “proposed by Tanaka et al. [10] were used”

Revised accordingly. Please see Line 217 in the revised manuscript.

• Line 287 – Suggest changing “most pigs in the populations studies” to “most pigs in the population studies”

Revised accordingly.

• Line 320 - Suggest changing “D7 haplogroup which accounted” to “D7 haplogroup, which accounted”

Revised accordingly.

• Line 324 – Suggest changing “what [5] recognized” to “what Scandura et al. [5] recognized”

Revised accordingly.

• Lines 333-334 – Suggest changing “the previous researches.” to “the previous research.”

Revised accordingly. See Line 334 in the revised manuscript.

• Line 340 – Suggest changing “wild boars” to “wild boar”

Revised accordingly. Please see Line 339 in the revised manuscript.

• Line 361 - Suggest changing “contrast, domestic pigs in MSEA combined revealed” to “contrast, combined domestic pigs in MSEA revealed”

Revised accordingly. Please see Line 360 in the revised manuscript.

• Line 404 - Suggest changing “Philippine pigs” to “Philippine domestic pigs”

Revised accordingly. Please see Line 403 in the revised manuscript.

• Line 437 - Suggest changing “Before the European arrived in the Philippines” to “Before the Europeans arrived in the Philippines”

Revised accordingly. Please see Line 436 in the revised manuscript.

• Line 442 – Suggest changing “which exhibited” to “which is exhibited”

Revised accordingly. Please see Line 441 in the revised manuscript.

• Line 446 - Suggest changing “was minimal, which accounted for only” to “was minimal, accounting for only”

Revised accordingly. Please see Line 445 in the revised manuscript.

• Line 464 - Suggest changing “Chinese pigs, therefore this precluded the origin of these haplogroup” to “Chinese pigs, which precludes the origin of this haplogroup”

Revised accordingly. Please see Line 463 in the revised manuscript.

• Line 480 – Suggest changing “populations despite shared haplotypes could” to “populations, despite shared haplotypes, could”

Revised accordingly. Please see Line 479 in the revised manuscript.

• Lines 490-497 – What “Philippine wild pigs” are being referred to here? The Philippine wild pigs that Bill Oliver was referring to in the IBEX article cited are not Sus scrofa, and in fact are all separate species from either domestic or wild Sus scrofa. Without identifying the species of the Philippine wild pigs that you are referring to, I’m not sure of the value of this paragraph to the rest of the study.

Revised accordingly. This paragraph was deleted.

• Lines 510-511 - Suggest changing “In addition, [72] have previously discussed” to “In addition, Scandura et al. [72] have previously discussed”

Revised accordingly. Please see Lines 500-501 in the revised manuscript.

• Line 525 – Suggest changing “distributions which represent” to “distributions that represent”

Revised accordingly. Please see Line 515 in the revised manuscript.

• Line 560 - Suggest changing “reported by [54]” to “reported by Frantz et al. [54]”

Revised accordingly. Please see Line 550 in the revised manuscript.

• Line 566 – Suggest changing “exchange of pigs” to “exchange of domestic pigs”

Revised accordingly. Please see Line 558 in the revised manuscript.

• Line 574 – What is the name “native pig populations” referring to here, domestic or wild pigs? There are no “native” domestic pig populations in the Philippines.

Revised accordingly. We change “native” to “local”. Please see Line 567 in the revised manuscript.

Reviewer #4: This manuscript aimed to present the origin and distribution of Philippine domestic pigs based on partial mitochondrial D-loop sequences analysis. The results showed the genetic connection between the Philippine domestic and continental pigs, supporting the Neolithic-Austronesian expansion model and proposing the possible alternative migration route. The data and analyses presented in the manuscript are appropriate, and the results support the key messages. The sequences under accessions MN625805-MN625830 are searchable; however, the sequences under accessions MW924902-MW92973 could not be found in the GenBank database. I am reviewing another manuscript submitted to a different journal. In that manuscript, some pictures of pigs looked the same as all sub-figures in Fig. 2, except Fig. 2F. In addition, some contents in the introduction section of that manuscript were very similar, if not the same, as some parts of the introduction of this manuscript; for example, the content on Page 4, Line 96-103 starts with the words "To date." However, more Philippine pig samples were included, and some analyses applied on mitochondrial D-loop sequences in that manuscript differed from this manuscript. In addition, the conclusion in that manuscript could be considered as an answer of the future direction of this manuscript mentioned on Page 23, Line 567-569, that " a more thorough investigation should be conducted to relate the possible ancestral diffusion that occurred from MSEA to the Philippines via Sundaland.

The authors highly appreciate the positive comments and suggestions by the Reviewer and for considering our manuscript for publication.

With regards to the accessibility of sequences in the GenBank database, they are still being processed and will be released to the public once finalized. Submissions are not automatically deposited into the GenBank after being accessioned, ensuring first that it is free of errors or problems.

This manuscript was submitted in the bioRxiv (a preprint server) to accelerate the dissemination of our research. This submission makes our work citable even before the formal publication. Thus, this article has already the Digital Object Identifier (DOI) that will later link the preprint and the final version of the manuscript when it is officially published, including citations. As we all know, peer-review takes time and getting submission to publication can take several months or even a year. Moreover, as our Laboratory handles huge research projects which in any circumstance be published ahead of time, this article is well-cited and credited in all our successive submissions. As also stressed by some Reviewers, this is the first study that has specifically looked at genetic composition relating to the anthropogenic origin of domestic pigs in the Philippines. This study is considered a baseline study and, therefore, a hot topic in the field. However, this manuscript still has an underlying question that needs to be addressed (e.g., stressed on Page 23, Lines 567-569). The other manuscript that we recently submitted is a confirmatory and a continuation study about this paper (as likewise, the Reviewer mentioned in his comments), which allows us to address the limitations of this article on that manuscript. Please see the new Figure 2 in the revised manuscript. Thank you very much for this clarification and the authors appreciate the Reviewer.

Despite the concerns in the previous paragraph, the following comments should be addressed before accepting publication.

Page 2, Line 61-62: Regarding the sentence on these lines, what is the population showing sudden decline? The population name, such as Philippine pigs, Mainland Southeast Asian pigs, should be specified. In my opinion, both Bayesian skyline plots (Fig. 7) did not clearly show a sudden decline, so I am wondering what result supported this statement? This comment also applied to the statement "The sudden population decline occurred approximately 25,000 years before present (BP)" on Page 15, Line 377-378 and "The underlying sudden population decline as predicted in the BSP markedly followed the LGM period" on Page 23, Line 571-572.

Revised accordingly. The word “Philippine domestic pigs” was added to these sentences. The authors are pointing out the BSP for Philippine samples. As the Figure projects, the BSP trend showed a stationary period of maternal effective population size however, the decline trend can be observed approximately 25,000 years BP which compliments the multimodal mismatch distribution of the Philippine samples. However, after further deliberation by all the authors and to avoid confusion, we decided to delete the word “sudden” in these sentences.

Page 8-9, Line 214-216: The statement "This method calculates the net divergence of each taxon from all other taxa as the sum of the individual distances from variance within and among groups of Philippine pigs and comparison sequences." should be revised. It was not whether the net divergence of each taxon was the sum of the individual distance, and the distance was calculated from within and between groups variance. In addition, is the term "variance" the statistical variance or the sequence differences?

Revised accordingly. Revised to “This method calculates the net divergence of each taxon from all other taxa as the sum of the individual distances from variance within and among groups.” We are referring the statistical variance. Please see Lines 214-216 in the revised manuscript.

Page 11, Line 262-264: How many haplotypes were found in total? The summation of the number of PHL, Cambodia, Bhutan, Laos, Myanmar, and Vietnam haplotypes was 74, but the sentence on Line 264 stated that "these sequences collapsed when pooled from 76 to 57 haplotypes.
Page 11, Line 265: Would the 26 haplotypes be found in 26 samples? If this is correct, should "a single sequence" be changed to "a single sample"?

Revised accordingly. Please see Lines 264- 265 in the revised manuscript.

Page 12, Line 285: The (Figs 2 and 3) should be changed to (Fig. 3 and 4).

Revised accordingly. Please see Line 285 in the revised manuscript.

Page 14, Line 335-337: Why would the presence of unique haplotypes, such as PHL21, in Philippine wild pigs suggest the presence of new subspecies of wild pigs? I think carrying the distinct mtDNA haplotype defined based on partial D-loop sequence is insufficient to suggest that the sample is the new subspecies.

Revised accordingly. This sentence was deleted, as likewise suggested by the first Reviewer. Thank you very much.

Page 16, Line 409-412: What was the result supporting the statement "The distribution of the haplotype frequencies did not indicate equilibrium."? In addition, would the genetic connection to the continental pigs shown by phylogeny and haplotype network suggest the multiple ancestors of Philippine domestic pigs?

The authors referred to the distribution of the shared haplotypes that are not equal. As based on the distribution, there are some haplotypes that are inter-island and regionally shared. For question number 2, we based this statement as clearly indicated by literatures that Eurasian wild boar is not native to the Philippine. Further, our analysis of the contemporary samples showed closed affinity and similarity in the mutational motif to those continental wild and domestic pigs, as well as where the fossil records where the major domestication took place.

Page 18, Line 447-449: The citation should be added to the sentence "This observation is consistent with the situation in domestic pigs in the MSEA, where the mtDNA of European pigs has a negligible impact on the maternal origin of domestic pigs."

Revised accordingly. Please see Line 447 in the revised manuscript.

Page 20, Line 494-497: Due to the limited sequences available and included in this study, do you have enough evidence to suggest that the Philippine wild pigs were descended from the indigenous wild pigs?

Revised accordingly. This paragraph was deleted as also suggested by Reviewer 1.

Page 47: Would it be possible to expand the network on Fig. 4, especially for haplogroup D2 and D7, to present the connection between haplotypes?

Revised accordingly. Please see Figure 4.
