# Supplementary material for: Insights on the historical biogeography of Philippine domestic pigs and its relationship with continental domestic pigs and wild boars
Source: PLoS One. 2022 Mar 28;17(3):e0254299. doi: 10.1371/journal.pone.0254299 (PMC8959178; doi:10.1371/journal.pone.0254299)
Supplement: Attachment — Submitted filename: Response to Editor.docx [file pone.0254299.s011.docx]

Line 55:  "collapsed with" is unclear. If the intent is to highlight the prevalence of D7 haplogroup in the Philippines perhaps "included" would be more appropriate.

Revised accordingly.

Lines 63-64: Which finding highlights an important challenge managing Philippine pig genetic resources and what is the challenge?

One of the reviewers recommended to add concluding statement in the abstract. However, to be more specific, we have changed this sentence to “The results of this study will support the conservation strategies and improvements of economically important genetic resources in the Philippines.”

Lines 202-204: The description of Arlequin AMOVA fixation indices is inaccurate and needs to be revised. As noted in the Arlequin user manual (Excoffier et al. 2005), PhiST tests for deviations from panmixia within the full sample, PhiCT tests the significance of designated population groupings, and PhiSC tests for differences among populations within groups.

Excoffier, L., Laval, G., & Schneider, S. (2005). Arlequin (version 3.0): an integrated software package for population genetics data analysis. Evolutionary bioinformatics, 1, 117693430500100003.

Revised accordingly. Please see Lines 200-201 in the revised manuscript.

Lines 220-252: My feeling is that population demographic analyses as presented may be inaccurate because groupings include historically isolated populations. This violates model assumptions and can bias results by reflecting changes in the degree of structure rather than changes in population size (eg. Heller et al. 2013). Given this, I think the impact of the paper could be improved by leaving out demographic analyses and placing the focus more clearly on phylogenetic and phylogeographic results. However, if you decide to retain demographic analyses please include a caveat highlighting the potential impact of historic population subdivision on demographic estimates.

Heller, R., Chikhi, L., & Siegismund, H. R. (2013). The confounding effect of population structure on Bayesian skyline plot inferences of demographic history. PloS one, 8(5), e62992.

Revised accordingly. We have decided to retain the demographic analyses by adding caution on the impact of historic population subdivision on demographic estimates for the Philippine samples. We added the following sentences, “However, since the Philippine samples included historically isolated populations that may have caused a sound impact on the demographic estimates, it could also be that the result reflected here is merely the changes in the degree of structure rather than changes in the population size.” Please see Lines 542-545 in the revised manuscript.

The following citation was also added:

Heller R, Chikhi L, Siegismund HR. The confounding effect of population structure on Bayesian Skyline Plot inferences of demographic history. PLoS ONE. 2013;8(5): e62992.

Line 264-265: The sentence starting with "Twenty-six..." is unclear.

Revised accordingly. We changed to “Twenty-six (6 PH and 20 MSEA) of the 57 haplotypes were found only once among the sequences.”. Please see Lines 266-267 in the revised manuscript.

Line 284: Because PhiST reflects the average contribution of population subdivision to deviation from panmixia, significance is possible even if some populations comparisons are not significantly different. Given this, please change "at all geographic locations examined" to something like "indicating significant genetic structure among the geographic locations examined."

Revised accordingly. Please see Line 285 in the revised manuscript.

Table 2: Please revise the "source of variation" column and related notes below the table to more accurately reflect Arlequin fixation indices. Specifically, PhiSC is more accurately described as "among populations within groups".

Revised accordingly.

Table 3: Is pairwise Fst or PhiST presented here? If genetic distance between haplotypes was included in the analysis then PhiST would be correct.

The population pairwise F*_ST_* is the one presented in Table 3 and not the PhiST. The authors, however, acknowledged that our discussion on the methodology was confusing. Please check the revised methodology, RE: Phylogenetic and population structure analysis. Lines 195-206 in the revised manuscript.

Line 333: "nomenclature quotation" is unclear.

Revised accordingly. Please see Line 341 in the revised manuscript.

Line 507: Is "Fst" a typo? If not, some clarification in the methods is needed.

Our apology for the confusion. This is not a typo. Please check the revised methodology, RE: Phylogenetic and population structure analysis.

Line 507: "individuals" seems to be a typo. Please correct.

Revised accordingly. Changed from “individuals” to “population”. Please see Line 500 in the revised manuscript.

Figure S1: There are no indels so the definition of the "minus" symbol can be deleted

Revised accordingly.

Figure S1: D7 is not included in the figure.

Revised accordingly. Please see the revised Figure S1.

Figure 1: Please enlarge the haplotype key to ensure readability.

Revised accordingly. Please see the revised Figure 1.

Figure 5-7: If demographic analyses are retained these figures should be moved to Supplemental data.

Revised accordingly. These Figures were moved to the supplemental data.
