# Supplementary material for: Insights on the historical biogeography of Philippine domestic pigs and its relationship with continental domestic pigs and wild boars
Source: PLoS One. 2022 Mar 28;17(3):e0254299. doi: 10.1371/journal.pone.0254299 (PMC8959178; doi:10.1371/journal.pone.0254299)
Supplement: Attachment — Submitted filename: Response to Editor.docx [file pone.0254299.s012.docx]

Line 48- delete "a" in "revealed a considerable"

Revised accordingly.

Line 50- add comma between "analysis with" AND delete comma between "(5.10%), harboring

Revised accordingly.

Line 55- delete "with"

Revised accordingly.

Line 59-61- This sentence is unclear and should be revised.

Revised accordingly. Revised to “The analyses of mismatch distribution and neutrality test were consistent with the Bayesian skyline plot which showed a long stationary period of effective population size.”

Line 132- delete "even"

Revised accordingly. Please see Line 128 in the revised manuscript.

Line 202- delete "namely"

Revised accordingly. Please see Line 198 in the revised manuscript.

Line 212- what was Fst used for pairwise analyses while PHIst was used for AMOVA? If a mutational model was included in pairwise comparisons then PHIst should be used. If genetic distance was not included then Fst is the accurate parameter, but if that is the case then I think a brief justification is needed to help readers understand why genetic distance was included in one set of analyses and not the other.

Our apology for the confusion. Unlike in AMOVA, where we calculated the standard “variance” components of each hierarchic characteristic at 3 levels: among groups defined a priori, among populations within groups, and among localities within populations ([Excoffier et al., 1992](javascript:;)), the population pairwise F_ST,_ on the other hand was performed to estimate the overall genetic divergence between two populations (i.e., BTN vs. KHM; VNM vs. PHL; etc.). The AMOVA here was done only to estimate population differentiation of the geographic divisions of the four genetic structure hypotheses.

Line 297- is "(Fst values, p<0.01)" a typo? Did you mean to include the range of Fst values here?

Yes, this is a typo. Revised accordingly. Please see Line 289 in the revised manuscript.

Line 336- delete "A" in "A widespread Asian ancestry"

Revised accordingly. Please see Line 322 in the revised manuscript.

Line 422- "underlies as a genetic basis" is unclear. Please revise

Revised accordingly. Please see Line 393 in the revised manuscript.

Line 427- what "equilibrium" are you referencing?

The authors referred to the distribution of the shared haplotypes which are not equal. As based on the distribution, there are some haplotypes that are inter-island and regionally shared.

Lines 435-437- Please revise for clarity and grammar. For example... "Thus, these events may have paved the way for the introduction of multiple distinct lineages of domestic pigs to the Philippines, as has been documented for chickens [56] and goats [57]."

Revised accordingly. Please see Lines 407-408 in the revised manuscript.

Lines 443-446- Please revise for clarity and grammar.

Revised accordingly. Revised to “The complexity surrounding the Neolithic Austronesian expansion and dispersal has recently raised several questions due to the limited genetic studies conducted in the Philippine archipelago, as the faunal assemblages in this key region have provided some clues [4].” Please see Lines 414 to 416 in the revised manuscript.

Line 446- replace "the preliminary studies that revealed" with "preliminary genetic studies revealed"

Revised accordingly. Please see Line 417 in the revised manuscript.

Line 449- add "in the Philippines" after "D2 haplotypes"

Revised accordingly. Please see Line 421 in the revised manuscript.

Line 458- change "precisely evident as shown by" to "supported by"

Revised accordingly. Please see Line 429 in the revised manuscript.

Line 461-  change "a crucial" to "an important"

Revised accordingly. Please see Line 431-432 in the revise manuscript.

Lines 471-474- This sentence is unclear, please revise.

Revised accordingly. Revised to “. Previously, it was emphasized that European pigs possessed both Asian and European mtDNA resulting from the extensive history of interbreeding with predominantly Asian mtDNA introgression [62-63,3], which currently accounts for approximately 20-35% Asian contribution to western modern breeds [64-66].” Please see Lines 442-445 in the revised manuscript.

Line 500- replace "and the consequent" with ", and subsequent

Revised accordingly. Please see Line 470-471 in the revised manuscript.

Lines 500-503- This sentence is unclear, please revise.

Revised accordingly. Rephrased to “In addition, as predicted by the theory of genetic isolation by distance, population differentiation usually occurs when there is migration of a certain population away from its founder population, leading to a reduction in genetic diversity.” Please see Lines 471-475 in the revised manuscript.

Line 503- is "founder" a typo? If I understand your point then "founder event" would be more accurate.

Revised accordingly. Please see Line 475 in the revised manuscript.

Line 504- "population" should be plural

Revised accordingly. Please see Line 475 in the revised manuscript.

Line 568- "sound impact" is unclear, please revise.

Revised accordingly. Please see Line 539 in the revised manuscript.

Line 569- delete "the" before "changes"

Revised accordingly. Please see Line 540 in the revised manuscript.

Line 570- delete "the" before "population size"

Revised accordingly. Please see Line 541 in the revised manuscript.

Line 578-580- This sentence is unclear, please revise.

Revised accordingly. Revised to “This study provided important insights that will properly help address the contradicting hypothesis of a possible human-mediated translocation and exchange of domestic pigs in the Philippines.” Please see Lines 549-551 in the revised manuscript.

Line 583- delete "flow"

Revised accordingly. Please see Line 554 in the revised manuscript.
